# Optical super-resolution histology of formalin-fixed paraffin-embedded tissue samples: challenges and opportunities

**DOI:** 10.1038/s41467-025-64626-1

**Published:** 2025-11-05

**Authors:** Luis E. Villegas-Hernández, Vishesh K. Dubey, Ganesh Acharya, Balpreet Singh Ahluwalia

**Affiliations:** 1 Department of Physics and Technology, UiT The Arctic University of Norway, Tromsø, Norway; 2 Division of Obstetrics and Gynecology, Department of Clinical Science, Intervention and Technology, Karolinska Institute, Stockholm, Sweden; 3 Department of Physics, University of Oslo, Oslo, Norway

**Keywords:** Microscopy, Fluorescence imaging

## Abstract

This review covers the advancements of optical super-resolution microscopy (SRM) on formalin-fixed paraffin-embedded (FFPE) histological samples. We cover the implementation of various SRM strategies in histology, including wide field methods such as structured illumination microscopy, single-molecule localization microscopy and fluorescence fluctuations-based SRM, as well as the point-scanning stimulated emission depletion microscopy. We also cover the recent developments in FFPE-based expansion microscopy. The review highlights the advantages and challenges of these SRM methods in FFPE histology, and provides insights into emerging optical and computational techniques that can potentially open avenues for understanding disease mechanisms, tailoring treatments, and advancing personalized medicine across disciplines. This review article is intended for a broad audience, including histopathologists, biologists, physiologists, and physicists.

## Abstract (web summary)

The review covers cutting-edge advancements in optical super-resolution microscopy (SRM) on formalin-fixed paraffin-embedded (FFPE) histological samples.

## Introduction

### Formalin-fixed Paraffin-embedded histology

*Histology* is the branch of science that studies the structure and organization of cellular and noncellular components of tissues using a microscope. Within it, *histopathology* focuses on analyzing the morphological and molecular alterations linked with the disease initiation and progression. In histological examinations, the sample preparation workflow varies according to the chosen microscopy technique. Yet, the common objective among the diverse preparation steps is to closely preserve the anatomical information of the tissue samples in their native state, to facilitate microscope visualizations. To this end, both the mechanical and biochemical stability of the tissue samples must be ensured.

**Fig. 1:**
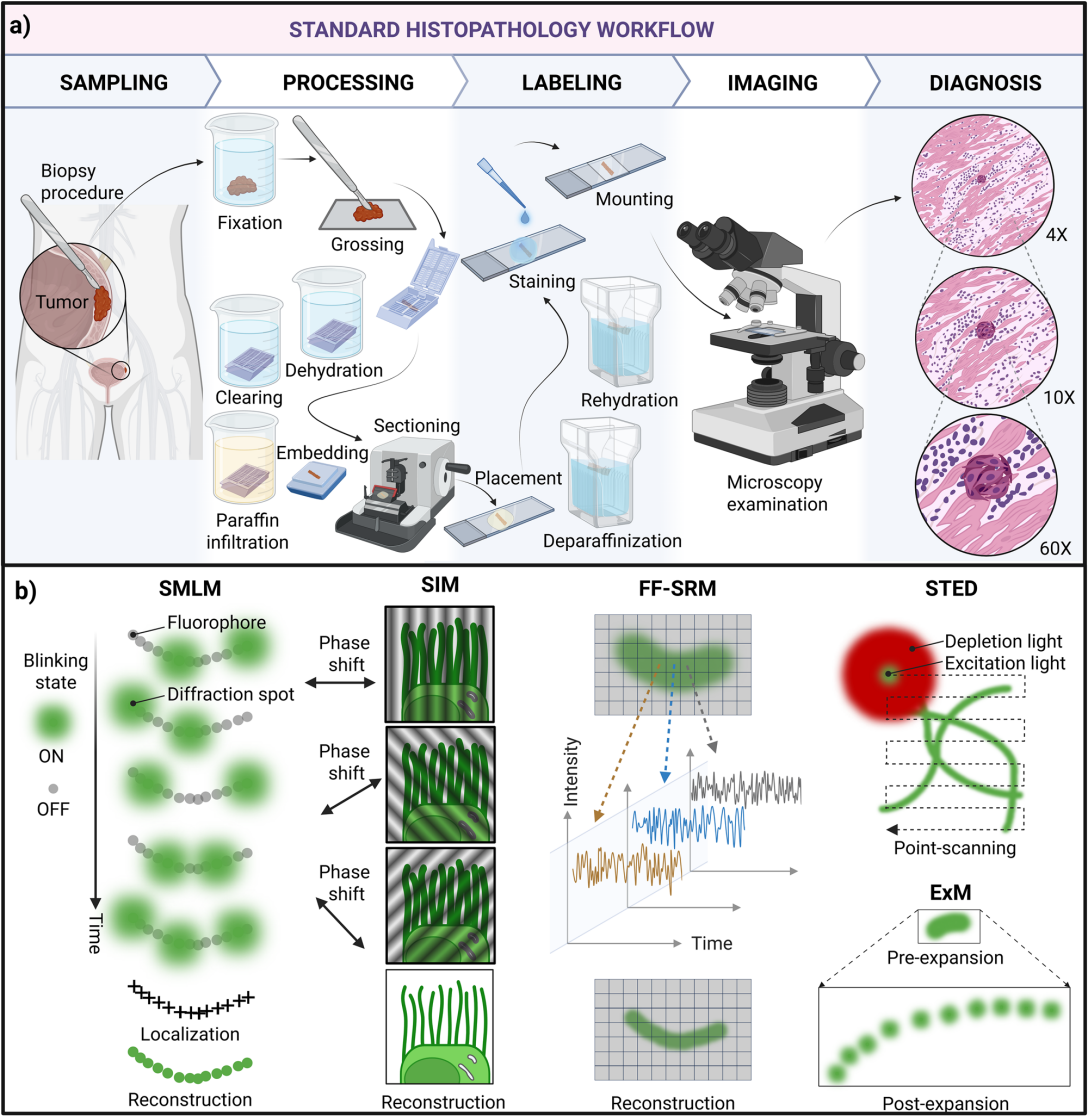
Schematic representation of diverse histological and super-resolution microscopy methods for the evaluation of formalin-fixed paraffin-embedded tissue samples. **a** A standard histopathological workflow involves multiple sequential steps, starting with extraction from the diseased organ, fixation in formalin, grossing, dehydration, clearing, paraffin infiltration, and embedding. The prepared tissue block is then sectioned on a microtome into thin slices (typically 2–5 µm thick). Subsequently, the tissue section is placed on a glass slide for further deparaffinization, rehydration, and staining. After glass coverslip mounting, the slide is brought to an optical microscope for inspection. To find the relevant features for analysis, the sample is hierarchically imaged at different levels of magnification according to the desired level of detail necessary for diagnosis. While this methodology allows the identification of many cellular and subcellular features, the resolving capabilities of conventional optical microscopes are limited by diffraction, restraining the ultrastructural assessment of tissues. **b** Diverse fluorescence-based optical microscopy techniques have been proposed for the ultrastructural investigation of Formalin-fixed Paraffin-embedded tissue sections. These include: single-molecule localization microscopy (SMLM), where the temporal ON/OFF blinking sparsity of the fluorophores is exploited, enabling precise localizations of multiple emitters within a diffraction spot. In structured illumination microscopy (SIM), the sample is illuminated using interference fringes, down-converting the high-frequency components (associated with the fine structural information) that are beyond the microscope’s resolution limit into the observable frequency spectrum. In fluorescence fluctuation-based super-resolution microscopy (FF-SRM), the intensity fluctuations of neighboring pixels are statistically analyzed over time to estimate the location of the emitting fluorophores. Stimulated emission depletion microscopy (STED) utilizes a doughnut-shaped depletion beam to deterministically reduce the area from where the emission is collected. In expansion microscopy (ExM), the sample is physically enlarged using chemical treatments before observation in an optical microscope. This figure was created in BioRender. Villegas, L. (2025) https://BioRender.com/a3u6yf4.

A common method for histological analysis in light microscopy is the so-called formalin fixation and paraffin embedding^1,2^ (FFPE). In FFPE, biological tissues are infiltrated with paraffin wax to ensure structural stability during microtome sectioning. The process, as illustrated in Fig. 1a, starts by extracting a portion of the organ for examination. This procedure is performed via a tissue biopsy or resection. To prevent putrefaction and halt enzymatic activity within the tissue, the sample is immersed in a formaldehyde derivative, namely formalin. The immersion in formalin promotes chemical fixation via molecular cross-linking. The sample is then manually cut into smaller fragments by grossing, followed by dehydration, paraffin infiltration, and embedding into paraffin blocks. Once solidified, the paraffin-embedded sample is sectioned into thin slices on a microtome (usually, 2–5 µm thickness) and collected on a microscope glass slide. The tissue section undergoes deparaffinization and rehydration to enable labeling and subsequent imaging. Nowadays, most of these procedures are fully automated using tissue processors and stainers, enabling the routine preparation of tens to hundreds of histological slides per day.

FFPE provides a simple, reliable, and cost-effective method for preserving, sectioning, and archiving tissue specimens at room temperature for decades^2^. This makes it a preferred histological processing technique in light microscopy. Moreover, the FFPE processing approach accommodates a variety of labeling procedures, including light-absorbing dyes such as hematoxylin and eosin, immunohistochemical markers, and light-emitting fluorophores used in fluorescence microscopy. FFPE has become the leading histological preservation method, with millions of samples currently stored in biobanks around the globe^3,4^. FFPE sample preparation has become an indispensable resource for a wide variety of clinical studies using complementary methods, ranging from simple microscopy to advanced genomics^5,6^ and proteomics^7^ to aid in diagnosis^2^ and prognosis^8,9^ of diseases.

### High-resolution histopathology

Enhancing the spatial resolution of histopathological examinations can aid in more precise and early disease diagnosis by providing improved visualization of subcellular structures and tissue micro-environment. The use of high-resolution imaging systems in pathology is driven by the growing need for understanding the molecular and structural changes associated with diseases. Genomic and epigenomic disruptions lead to alterations in nuclear architecture and other subcellular organelles throughout all stages of disease progression^10,11^. Consequently, structural transformations within cellular and tissue structures are expected to evolve gradually, from subtle to significant changes^12^. In recent decades, the usage of fluorescence-based imaging modalities has become popular both in histology and histopathology. This includes classical optical techniques such as epifluorescence, confocal, and the more recently developed super-resolution microscopy (SRM)^10,13–16^. The main advantage of these techniques is their ability to deliver high specificity and high-resolution feature detection of cells and tissues^17^.

In conventional optical microscopes, the lateral resolution is limited by the diffraction of light to about 250 nm, which can impede both accurate histological analysis and disease diagnosis^10^. Therefore, these are often referred to as diffraction-limited systems. While many microscopic features can be observed at this resolution, the ultrastructural architecture of tissues falls beyond the resolving capabilities of conventional optical microscopes. Hence, features like tight junctions, synapses, foot processes, and microvilli brush-border, as well as diseases such as minimal change disease^18^, primary ciliary dyskinesia^19^, amyloidosis^20^, among others, necessitate the electron microscopy (EM) for visualization. The current histopathological workflow in these cases involves both contextual tissue sample observations over large fields-of-view (FOV) by optical microscopy and nanoscale visualizations of selected sample areas by electron microscopy. This procedure not only implies two different sample preparation protocols and two distinct microscopy imaging methods, but also a lengthy, costly, and suboptimal process. While both high-resolution and high-throughput are essential, other aspects are equally important in clinical practice. These include the ease of sample preparation, compatibility with standard histopathology pipelines, and affordability.

Although EM provides ultra-high resolution, it comes with a cost of lengthy sample preparation, a lack of compatibility with standard tissue sections, low imaging throughput, low specificity and elevated operational costs. On one hand, the sample preparation for transmission electron microscopy (TEM) involves several steps, including dehydration, resin embedding, ultrathin sectioning, and heavy metal staining, which are time-consuming and technically demanding. On the other hand, standard scanning electron microscopy (SEM) can only provide information on the 2D surface morphology. While modern SEM methods are available for 3D imaging, they are slow, complicated, and costly; therefore, not scalable for routine histopathology. Hence, a single microscopy technique offering both high-speed diffraction-limited imaging and super-resolution imaging, which can be integrated within standard histopathological pipelines, will streamline clinical assessment. To this end, it is attractive to explore the use of fluorescence-based SRM in histopathology, as the same instrument can typically be operated in diffraction-limited microscopy mode to scan large FOVs, while the SRM modality can be utilized for higher resolution imaging of the regions of interest. SRM integrates nanometer-scale resolution with a high molecular specificity^21^, making it a powerful tool for tackling the complex challenges in histopathology. SRM typically uses standard immunofluorescence staining protocols that are already common in pathology labs. This can reduce costs and complexity for health institutions, while alleviating patient burden.

## Super-resolution histology of Formalin-Fixed Paraffin-Embedded Tissues

Over the last two decades, an innovative set of optical microscopy techniques has been developed to achieve sub-diffraction resolution, surpassing the lateral resolution limit of 250 nm. The key to breaking the diffraction limit was by exploiting the photokinetic properties of fluorophores and by conducting laser beam engineering. Referred to as fluorescence-based SRM, this set of techniques allows nanoscale visualization of biological tissues without preparing them for electron microscopy. The SRM methods have revolutionized the field of life sciences, offering several advantages, including high contrast, high specificity, and high resolution. The SRM methods are broadly divided into five categories, four of which follow a far-field optical microscopy approach, and one based on a biophysical expansion of the specimen. These include:

- **Single-molecule localization microscopy (SMLM)**^22^, which maps the coordinates of individual molecules by exploiting the temporal sparsity via ON/OFF fluorophore blinking;
- **Structured illumination microscopy (SIM)**^23^, which uses a periodic structured illumination onto the sample to create a sample-illumination spatial frequency mix containing sample details beyond the observable limits of the imaging objective lens. Thereafter, the super-resolution information is extracted by using a mathematical reconstruction algorithm;
- **Stimulated emission depletion microscopy (STED)**^24^, which utilizes a deterministic approach to decrease the size of the emission spot by using a doughnut-shaped depletion beam;
- **Fluorescence fluctuation-based super-resolution microscopy (FF-SRM)**^25,26^, where the collective signal fluctuations of neighboring pixels are statistically analyzed over time to estimate the location of the individual fluorescence emitters.
- **Expansion microscopy (ExM)**^27^, which, in contrast to the previous far-field methods, supports the visualization of nanoscale features by isotropically enlarging the biological specimens through the embedding and physical expansion of a hydrogel reagent.

Figure 1b offers a schematic representation of the SRM methods listed above. For a comprehensive overview of these methods, the reader is referred to the existing SRM review literature^28–31^.

### Applications of super-resolution microscopy in Formalin-Fixed Paraffin-Embedded histology

The development of SRM has demonstrated its potential to advance both clinical and research applications, allowing for detailed visualization of tissue sections beyond the reach of traditional microscopy methods. SRM methods have transformed histopathology by enabling the visualization of nanoscale subcellular structures that are crucial for accurate detection, diagnosis, and understanding of tissue anatomopathologies^4,10,32,33^. Recent advancements in microscopy techniques and computational algorithms have significantly enhanced the resolution, imaging area, and throughput, improving the potential of histopathological diagnosis and histological analysis^4,10,32–34^. As summarized in Table 1, to a greater or lesser extent, all the SRM methods have been implemented on FFPE samples. A summary of these implementations is presented here.

**Table 1:** Summary of super-resolution microscopy methods applied for Formalin-fixed Paraffin-embedded histology

SRM method	Histological application	Tissue type	Research outcomes	Other comments	Ref. #
SMLM	Cancer research	Human breast	Nanoscale assessment of mitochondria, nuclear membrane, and HER2 receptor. 2D and 3D visualizations.	FOV ∼50 × 50 µm^2^. Large reconstruction dataset	^4^
	Histopathology	Mouse intestine, Human colon	SMLM workflow optimization for high-throughput reconstruction of high-density emitter datasets.	FOV ∼30 × 30 µm^2^	^34,35^
	Cancer research	Human colon	Quantification of structural changes in the chromatin content of colorectal cancer.	FOV ∼30 × 30 µm^2^. Large reconstruction dataset	^36,37^
	Cancer research	Human pancreas	Large FOV visualization of the mitochondrial structure via prism-based TIRF illumination. Color multiplexing.	Large reconstruction dataset	^38^
	Neuronal research	Human & Mouse brain, spinal cord	Localization of aquaporin-4 clusters in astrocytic processes of brain and spinal cord samples. Correlation with neuronal diseases. Dual color imaging.	Large reconstruction dataset	^39^
	Neuronal research	Human brain	Study of synapse interactions in brain samples from healthy and Alzheimer’s cases.	FOV up to ∼40 × 40 µm^2^	^40,41^
	Kidney research and diagnostic	Mouse and human kidney	Protocol for routine SMLM imaging of renal tissue for focal segmental glomerulosclerosis and minimal change disease.	FOV ∼50 × 50 µm^2^. Large reconstruction dataset	^43^
SMLM, SIM, STED, ExM	Kidney research	Mouse kidney	Visualization of the slit diaphragm meandering pattern using diverse SRM methods. Practical comparison.	Large reconstruction dataset for SMLM. Small FOV in SIM and STED. Specialized sample preparation for ExM	^42^
SIM	Ophthalmic research	Human eye retina	Study of macular degeneration by analyzing the autofluorescent signal from lipofuscin aggregates in the retinal pigment epithelium.	FOV ∼40 × 40 µm^2^	^44,45^
	Histopathology	Human & Mouse kidney	Identification of differences between podocyte processes of diseased and healthy specimens associated with glomerulopathies. 3D imaging. Multicolor.	FOV ∼50 × 50 µm^2^	^46–48,49^
	Kidney research	Human & Mouse kidney	Quantitative study of mitochondrial networks in kidneys. Morphogenesis of acute kidney injury. 3D imaging.	FOV ∼30 × 30 µm^2^	^50^
	Perinatology research	Human placenta	High contrast visualization of subcellular features in chorionic villi tissue.	FOV ∼40 × 40 µm^2^	^51^
STED	Kidney research	Human and Mouse kidney	Quantification of morphological features associated with glomerular disease predisposition in control and genetically modified mouse models. Dual color 2D imaging.	Point-scanning imaging. FOV ∼200 × 200 µm^2^	^52^
	Histopathology	Human & Mouse kidney	Quantification of differences in podocyte morphology between healthy and diseased samples. 3D dual color imaging.	Point-scanning imaging. FOV ∼100 × 100 µm^2^	^53^
	Dermatology research	Human skin	Quantification of nanoscale differences in molecular marker distribution of nPI(4,5)P2 between healthy and HPV-induced wart epidermal samples. Multicolor 2D imaging.	Point-scanning imaging. FOV ∼10 × 10 µm^2^	^54^
	Cancer research	Human skin	Microenvironment distribution visualization of immune PD-1 checkpoint inhibitor in melanoma.	Point-scanning imaging. FOV ∼10 ×10 µm^2^	^55^
	Cancer research	Human rectum	Nanoscale observation of mitochondrial morphology in long-term stored HER2 positive colorectal samples.	Point-scanning imaging. FOV ∼200 × 200 µm^2^	^33^
	Neuronal research	Human brain	Identification of tau filaments associated with Alzheimer’s disease.	Point-scanning imaging. FOV ∼15 × 15 µm^2^	^56^
FF-SRM	Histopathology	Human & Mouse, skin	Visualization and quantification of fibrotic-related changes in extracellular matrix over large FOV using autofluorescence.	FOV ∼320 × 340 µm^2^ over a 1.2 µm depth of field	^57^
	Histology research*	Human & rat, kidney and placenta	Visualization of ultrastructural features in human and rat kidney, and human placenta using autofluorescence in the label-free mode.	FOV ∼200 × 200 µm^2^	^59^
ExM + FF-SRM	Histology research	Human & Mouse, various	Quantification of diagnostic features in podocyte injury from human kidney biopsies. Ultrastructural visualization of different tissues.	Special sample preparation steps for expansion	^60^
	Histopathology	Human kidney	Visualization of ultrastructural features in various tissues, including colon, breast, uterus, placenta, thymus, thyroid, and kidney. Multicolor 3D imaging.	Special sample preparation steps for expansion	^61^
ExM	Histopathology	Human, various	Observations of nanoscopic features in clinical samples of diverse human organs using confocal and widefield fluorescence microscopes. Identification of minimal change disease, and early stages of breast cancer.	Special sample preparation steps for expansion	^32^
	Histopathology	Human kidney	Identification of renal diseases requiring the visualization of tertiary podocyte foot processes.	Special sample preparation steps for expansion	^62,63^
	Histopathology	Human, various	Reduction of expansion processing time from 2 d to less than 8 h. Demonstration on various FFPE tissues.	Special sample preparation steps for expansion	^64^
	Histopathology	Human & Mouse, various	Ultrastructural visualization of features in diverse tissues by labeling of carbohydrates and amines.	Special sample preparation steps for expansion	^65^

*HER2* human epidermal growth factor receptor 2, *HPV* human papillomavirus, *nPI(4,5)P2* nuclear phosphatidylinositol 4,5-bisphosphate, *PD-1* programmed cell death 1, *SRM* Super-resolution microscopy, *SMLM* single molecule localization microscopy, *SIM* structured illumination microscopy, *FF-SRM* fluorescence fluctuations-based super-resolution microscopy, *STED* stimulated emission depletion microscopy, *ExM* expansion microscopy, *Label-free super-resolution microscopy.

Starting with SMLM, this method has been used for the nanoscale assessment of breast cancer to identify morphological features beyond the resolving power of conventional optical microscopy, including the mitochondria, the nuclear membrane, and the HER2 receptor clustering^4^. Also, diverse SMLM-based approaches have been developed to study gastrointestinal neoplasia^34,35^, facilitating detection of morphological changes in the chromatin content of colorectal cancer^36,37^. More recently, a prism-based approach has been successfully implemented for large FOV visualization of mitochondrial structure in pancreatic tumors via SMLM^38^. Apart from cancer, the SMLM method has been implemented in neuronal research both for the localization of aquaporin-4 protein clusters^39^ in astrocyte  processes of brain and spinal cord tissues, and for the study of synapse interactions in brain samples of Alzheimer’s disease^40,41^ derived from human patients. Moreover, the SMLM methodology has been implemented to study the slit diaphragm architecture in the glomerulus of mouse kidney sections^42^, and its feasibility for renal studies was further demonstrated in human and animal samples^43^.

Similarly, the SIM method has been implemented for the visualization of diverse histological samples. For example, SIM has been proposed for the study of macular degeneration by analyzing the autofluorescence signal stemming from lipofuscin aggregates in the retinal pigment epithelium of human eyes^44,45^. More broadly, the SIM methodology has shown promising applications for the assessment of glomerulopathies in kidney samples from animal and human origin by allowing the identification of morphological differences between podocyte processes of diseased and healthy specimens^42,46–49^. Similarly, SIM has been proposed for the study of mitochondrial networks in mouse and human kidney tissue sections, shedding light on the morphogenesis of renal diseases such as acute kidney injury^50^. Next, in the field of perinatology, the high-resolution and high-contrast images supported by SIM have been used for visualizing subcellular features of chorionic villi in human placental sections^51^.

STED microscopy, on the other hand, has been employed for the visualization of ultrastructural features in renal samples, including the identification of the nephrin protein in the filtration barrier of mouse kidney^42^, and the quantitative study of morphological features associated with glomerular disease in genetically modified mouse models^52^. Also, the STED methodology has been successfully implemented for the three-dimensional assessment of human kidney, enabling the quantification of structural differences in podocyte morphology between healthy and diseased samples exhibiting focal segmental glomerulosclerosis^53^. STED has also been implemented for the study of human skin pathologies, including the quantitative identification of nanoscale differences between healthy and papillomavirus-induced wart skin samples in humans^54^, as well as the visualization of the microenvironment distribution of immune checkpoint inhibitor PD-1 in skin samples of melanoma^55^. The STED method has been successfully implemented for the nanoscale observation of mitochondrial morphology in HER2-positive human colorectal cancer samples stored at room temperature for 17 years^33^, hence demonstrating the advantage of paraffin-embedding for the preservation of ultrastructural information over elongated periods. Lastly, STED microscopy has been proposed for the identification of tau filaments associated with Alzheimer’s disease, which assists in decoding the mechanisms of pathology progression in tauopathies^56^.

When it comes to FF-SRM, a recent study demonstrated its applicability for visualizing the extracellular matrix in fibrotic diseases of mouse and human tissue by exploiting autofluorescence signals from collagen fibers^57^. In another study, researchers demonstrated the capabilities of the chip-based microscopy methodology^58^ for label-free super-resolution imaging of kidney and placental tissues^59^. Apart from this, two studies combined ExM (expansion microscopy) and FF-SRM methodologies to successfully reconstruct ultrastructural features in various human and animal tissues, reaching down to 15–25 nm lateral resolution^60,61^.

In recent years, the ExM approach has gained significant attention in FFPE-based super-resolution histology. Particularly, the development of an ExM variant termed expansion pathology (ExPath) has enabled 70 nm observations of anatomical features in clinical samples of diverse human organs (e.g., prostate, lung, breast, etc.) using confocal and widefield fluorescence microscopes^32^. Similarly, the ExPath method has proven useful for the identification of nephropathies (minimal change disease) and in early detection of different cancer types. Several studies have also proposed the ExPath method as a viable route for the diagnosis of renal diseases requiring the visualization of tertiary podocyte foot processes^42,62,63^. Moreover, recent efforts have shortened the expansion protocol from 2 days to less than 8 h, enabling a rapid version of the ExPath method (rExPath) that is compatible with routine histological timeframes^64^. Another approach, termed FLARE^65^ (fluorescent labeling of abundant reactive entities) makes use of the MAP (magnified analysis of the proteome) expansion methodology^66^ to offer high-detail contextual visualizations of ultrastructural features in samples of diverse tissues (e.g., kidney, liver, intestine, prostate, etc.) by covalently labeling chemical functional groups such as carbohydrates and amines.

### Challenges and opportunities

Despite the successful implementation of SRM in FFPE-based histology, several barriers hinder its widespread adoption in routine clinical and research settings. These include lower imaging throughput compared to conventional optical microscopy, susceptibility to optical aberrations, sensitivity to high labeling densities, reconstruction artifacts, methodological complexity, and the need for specialized sample preparation. The limitations of each SRM method for FFPE-based histology are presented here:

- **Single-molecule localization microscopy**: Albeit the robustness, versatility, and -to some extent- simple instrumentation of the SMLM methods, their penetration in routine histology remains particularly challenging due to the demanding requirements of the SMLM reconstruction process. These include the need for high photon emission, low background signal, low labeling density, high illumination intensities (particularly in the *d*STORM^67^ variant), minimal sample-stage drift, low motion blur, and the right chemical formulation of the blinking buffers^22^. In addition, the paraffin-embedded samples exhibit high autofluorescence signal^68^, light scattering, and epitope masking^69^ that further complicate the application of SMLM approaches^35^. These factors hamper the localization precision of the SMLM methods by increasing both the background signal and number of false localization events, therefore requiring additional optimization steps such as bleaching^42^, optical clearing^36^, and antigen retrieval to visualize single-molecule blinking events^22^.
  In addition, the low imaging throughput further limits the adoption of SMLM in histological practice. Particularly, when a large number of frames, typically between 30,000 and 70,000, are required to reconstruct a single super-resolved image using SMLM methods. The entire imaging process may take several tens of minutes to hours, even after using modern scientific cameras and fast processing computers. Moreover, several commercial applications of SMLM employ high-magnification and high numerical aperture microscope objectives^70^, leading to limited FOVs of around 50 µm × 50 µm. Although unique illumination approaches have boosted the SMLM imaging throughput for the visualization of cells and bacteria by exceeding FOVs of 100 µm × 100 µm using objective-based illumination^71,72^, or even up to the millimeter scale via de-coupled total internal reflection fluorescence (TIRF) illumination^38,73^, the evaluation of commonly available FFPE histological samples via SMLM methods still represents a labor-intensive task. This is mainly due to the large lateral dimensions of the tissue samples, which span up to the centimeter scale, thus demanding multiple rounds of SMLM acquisition and reconstruction to visualize the sample context^38^. Hence, the implementation of SMLM methods onto conventional FFPE samples could be deemed not only time-demanding but also technically costly in terms of computational resources and storage infrastructure.
- **Structured illumination microscopy**: SIM holds strong potential for histological applications that need high speed and high-throughput imaging, such as in intraoperative diagnosis^74^. However, commercial implementations of SIM are commonly limited to FOVs of about 50 µm × 50 µm, therefore requiring tile-mosaic imaging approaches to achieve large FOVs in histological samples^74,75^. Although recent approaches such as transmission-SIM^76^ and fiber-SIM^77^ have demonstrated extended FOVs beyond 150 µm × 150 µm, the applicability of these methods to FFPE-based histology has not yet been demonstrated.
  Another limiting factor for the adoption of SIM in histology is its susceptibility to optical aberrations^78^. Being a computational method, SIM is prone to image reconstruction artifacts caused by suboptimal imaging parameters and optical imperfections caused by system misalignment or by the specimen itself. For FFPE tissue sections, this challenge is pronounced due to refractive index heterogeneities arising from a sample-mounting media mismatch. Moreover, the 3D-SIM algorithms are sensitive to asymmetries in the optical point spread function stemming from light scattering of thick specimens, and to the intensity variations of the fluorophore emission during image acquisition, owing to photobleaching. This problem is further exacerbated by the autofluorescence nature of the FFPE tissue sections and by the background signal, which reduce the illumination fringe contrast,  making it difficult for the reconstruction algorithm to accurately determine the reconstruction parameters. Together, all these challenges reduce the quality of the SIM reconstructed image and could also generate pseudo-structures, potentially compromising its applicability in routine clinical analyses.
- **Stimulated emission depletion microscopy**: The point-scanning principle of this method makes it impractical for imaging the large areas (centimeter-scale) of the tissues, as required in histology. Moreover, dense regions of tissue samples induce light scattering aberrations, distorting the depletion laser shape and affecting the spatial resolution of the STED method^79^. To address these challenges additional clearing steps to homogenize the sample’s refractive index^42,53^ are used. Also, STED requires photo-stable fluorophores to withstand the high intensities of the depletion beam, making it incompatible with standard fluorescence labeling procedures.
- **Fluorescence fluctuation-based super-resolution microscopy**: Despite being an attractive route for high-throughput and high-resolution histological analysis, requiring only 30–100 frames for image reconstruction^26^, the implementation of the FF-SRM approaches in FFPE sections is primarily limited by the tight labeling confinement in tissues^57^ and the low fluorescence signal variability stemming from intrinsic fluorophore fluctuations^80^.
- **Expansion microscopy**: Undoubtedly, ExM has gained significant attention in life sciences, being often regarded as a method for democratizing SRM in life sciences^81^, owing to the use of conventional optical microscopes for sample assessment. However, the advantages of physically enlarging tissue samples come at the cost of specialized and manual sample preparation protocols that deviate from standard histological workflows, therefore deferring the adoption of this methodology in clinical practice.

Apart from the technical limitations of the SRM techniques, the FFPE methodology also poses challenges that affect sample preparation, microscopy, post-processing, and image analysis. During sample preparation, for example, the section thickness is a crucial parameter that must be carefully controlled. Particularly, thickness exceeding 5–10 µm can lead to poor light penetration and overlapping structures, while excessively thin sections of less than 3 µm may lose key morphological details by means of tearing and folding. Further, fixation artifacts, such as tissue shrinkage from dehydration or excessive crosslinking from over-fixation, can distort cellular structures and impact staining quality. The staining inconsistencies are another major challenge, including uneven or excessive staining, which further complicates the visualization of the morphological structure and potentially misleads the histological analysis. Additionally, improper mounting can introduce air bubbles or refractive index mismatches that distort imaging. In microscopy, light scattering and absorption, particularly in dense or pigmented tissues, reduce clarity, while autofluorescence from certain tissue components or fixatives can interfere with fluorescence imaging. Optical aberrations, result from refractive index mismatch and broadly affect the resolution, especially in thick sections. SRM techniques usually struggle with out-of-focus blur in thicker tissues, necessitating advancement in the imaging technique and post-processing algorithms. In image analysis, uneven illumination, low signal-to-noise ratio, and staining variability pose challenges for accurate quantification. Automated analysis can be affected by color inconsistencies across samples, requiring normalization techniques. Edge artifacts, lens distortions, and optical aberrations also impact the image quality, requiring computational corrections.

### High-throughput & label-free optical microscopy methods

In addition to fluorescence-based SRM, which provides both super-resolution and specificity, several demonstrations of high-resolution label-free imaging of FFPE tissue sections have been reported. Label-free optical microscopy methods such as lens-less holography, digital holography and Fourier ptychography (FP), albeit diffraction-limited, provide high-speed quantitative phase measurement capabilities^82,83^. FP enhances histopathological imaging by improving resolution over large sample areas, enabling the analysis of standard FFPE tissue sections at fine cellular details^84^. FP aids in detecting and quantifying morphological changes in unstained samples, improving tumor detection and grading through high-contrast imaging^85^. Further, the integration of FP-based quantitative phase imaging with artificial intelligence (AI) can potentially enable automated disease diagnosis by identifying the optical path length differences between benign and malignant cells in clinical slides^86,87^. Its potential to transform digital pathology lies in the ability to reduce laboratory costs (labeling supplies and overhead costs), streamline workflows, and enhance diagnostic accuracy^86^.

Another label-free methodology for histopathological analysis is the so-called non-linear optical microscopy, which combines second and third harmonic generation with two- and three-photon excited autofluorescence. Albeit demonstrated on FFPE samples^88^, this technique is emerging as a promising tool for real-time tissue diagnostics, allowing for high-resolution and high-speed imaging of fresh, unprocessed tissue without staining. To name a few examples, recent studies have demonstrated its effectiveness in the assessment of fresh breast and brain biopsies during surgery, closely resembling hematoxylin and eosin histology, as well as for in vivo screening of melanoma in skin lesions^89–91^.

Similarly, owing to the advancements in automation and data processing, the field of digital pathology has revolutionized routine FFPE-based histopathology by providing tools for imaging, storage, and diagnosis. Digital pathology is changing the way histopathologists analyze tissue sections, making the process faster, more efficient, and more accessible. The development of whole-slide imaging has made it possible to digitize entire tissue slides at high resolution, allowing pathologists to remotely access and share cases, surpassing the hurdles of shipping embedded samples and/or stained glass slides^92,93^. Moreover, high-speed scanners enable seamless integration with pathology databases and laboratory information systems, improving workflow efficiency and facilitating collaboration across institutions^92^. Additionally, the use of AI in digital pathology has enhanced diagnostic accuracy by enabling automated image processing, tumor detection, and biomarker quantification for prognostic markers such as HER2, Ki-67, and PD-L1^94–98^. AI-driven tools are also being used to pre-screen histopathological slides, assist in grading dysplasia, and improve the reproducibility of complex pathological assessments^99^. Despite these advancements, factors such as data storage and data management hinder the full-scale adoption of digital pathology in FFPE tissue analysis, as whole-slide images typically exceed hundreds of megabytes, requiring extensive IT infrastructure for efficient retrieval and long-term storage^92^.

To the best of our knowledge, only one study has reported label-free optical microscopy based on photonic-chip illumination, surpassing the diffraction barrier and achieving a lateral resolution of 144 nm in human kidney tissue^59^. The photonic chip-based microscopy opens an opportunity to develop a multi-modal optical microscopy platform that is not only compatible with standard histopathology pipelines, but also capable of performing high-speed quantitative phase imaging^100^ and auto-fluorescence fluctuation-based super-resolution imaging. Further, deep learning approaches, coupled with transfer learning strategies are generating super-resolution images from low-resolution biopsy slices, making high-quality imaging more accessible and cost-effective. These methods grasp transfer learning approaches to address the complexity and scarcity of biopsy image data, retaining critical texture details, making high-quality imaging more accessible to clinics and researchers^101–103^.

## Discussion

In this review, we have covered the latest implementations of SRM methodologies into FFPE-based histology, offering a global perspective of the challenges and opportunities of SRM to the research and clinical fields. We outline the latest applications of existing SRM techniques on standard FFPE sections, as summarized in Table 1. Despite the unprecedented levels of ultrastructural visualizations reported on the different SRM methods, we bring to notice that the widespread implementation of SRM methodologies in FFPE-based histopathology is still far from reality. This is evident by the relatively low number of published articles, fewer than 50 over the last decade, compared to the extensive life science literature involving SRM, with a current rate of nearly a thousand publications per year^28^. This disparity can be attributed not only to the optical challenges imposed by the FFPE tissue sections but also to the intrinsic limitations of the very SRM methods for routine histological analysis.

We presented an in-depth analysis of the latest efforts and elaborated on the technical and operational barriers that currently hinder the widespread integration of SRM into FFPE histological practice. First, slow imaging speed is a major challenge; most SRM techniques, STED, SIM, SMLM suffer from small fields of view and/or slow imaging speeds, making them inefficient for analyzing large tissue areas commonly required in histological assessments. Second, SRM methods are highly susceptible to optical distortions induced by the sample itself. FFPE tissues often exhibit refractive index heterogeneities and dense labeling, which degrade image quality; for example, SIM and STED are vulnerable to phase distortions, while SMLM can be significantly impaired by excessive fluorophore overlap. Third, the high operational costs associated with commercial SRM systems, including both capital investment and maintenance, remain prohibitive for many clinical and academic histopathology laboratories. Further, system complexity and the need for advanced user expertise represent a substantial hurdle. Techniques such as SIM and STED require complex setup, calibration, and data analysis, posing a steep learning curve for routine histopathological analysis. Finally, many SRM protocols demand non-standard sample preparations that diverge from conventional FFPE workflows. Methods like ExM, STED, and SMLM often involve enzymatic digestion, specific fluorophore selection, or physical sample expansion, introducing additional complexity into the histological pipeline. Taken together, these factors underscore the need for an improved microscopy platform that can deliver sub-diffraction resolution without compromising compatibility with existing FFPE sample processing and imaging workflows. We anticipate that the practical realization of super-resolution histology will require a combination of key features, including high spatial resolution, high throughput, low operational complexity, and cost-efficiency.

In addition to technical advancement in the fluorescence-based SRM techniques, the landscape of histopathological imaging is undergoing a profound transformation driven by the convergence of advanced optical microscopy methods and cutting-edge computational tools. While SRM offers unparalleled specificity and resolution, label-free, high-throughput methods such as Fourier ptychography are emerging as powerful, cost-effective alternatives for quantitative phase imaging in clinical settings. Despite being diffraction-limited, these techniques enhance diagnostic capabilities through high-speed imaging and compatibility with unstained or minimally stained samples, especially when integrated with AI for automated analysis. The shift toward digital pathology facilitated by whole-slide imaging, AI-assisted diagnostics, and cloud-based platforms is streamlining workflows, improving reproducibility, and enabling remote collaborations. However, challenges such as data management, algorithm validation, and standardization remain significant hurdles for widespread clinical adoption. Importantly, current implementations of label-free microscopy remain resolution-limited, highlighting a critical opportunity for the development of multi-modal platforms that integrate high-speed quantitative phase imaging with fluorescence-based super-resolution imaging. Recent advances in deep learning and transfer learning further support this vision by enabling the generation of super-resolved images from low-resolution images, thus democratizing access to high-quality histological imaging. The recent advances in deep learning and transfer learning are not only enhancing our diagnostic toolkit but also laying the groundwork for precision medicine, where real-time, AI-supported imaging could play a central role in disease detection, grading, and therapeutic decision-making. As innovation continues, the integration of optical and computational technologies holds immense promise to redefine the future of digital pathology, bridging the gap between traditional histology and next-generation diagnostics.

We contend that the successful implementation of SRM in FFPE-based histology could profoundly expand the frontiers of tissue analysis. In research settings, it could enable the discovery of nanoscale tissue architecture and molecular organization that remain inaccessible through conventional light microscopy. We envision that, particularly in clinical applications, the adoption of SRM histology would become a core modality in future histological and diagnostic practice, enabling the detection of subtle anatomopathological changes occurring at the nanoscale domain.
